# Vaginal Primary Malignant Melanoma: A Rare and Aggressive Tumor

**DOI:** 10.1155/2013/137908

**Published:** 2013-07-22

**Authors:** Georgios Androutsopoulos, Emmanouil Terzakis, Georgia Ioannidou, Athanasios Tsamandas, Georgios Decavalas

**Affiliations:** ^1^Department of Obstetrics and Gynaecology, University of Patras, Medical School, 26504 Rio, Greece; ^2^Department of Radiation Oncology, St. Savvas Anticancer-Oncologic Hospital, Athens, Greece; ^3^Department of Pathology, University of Patras, Medical School, 26504 Rio, Greece

## Abstract

Vaginal primary malignant melanoma is a rare and very aggressive tumor. It most commonly occurs in postmenopausal women, with a mean age of 57 years. Our patient is an 80-year-old, postmenopausal Greek woman presented with a complaint of abnormal vaginal bleeding. On gynecologic examination there was a pigmented, raised, ulcerated, and irregular lesion 5 × 4.5 cm in the upper third of anterior vaginal wall. She underwent a wide local excision of the lesion. The histopathology revealed vaginal primary malignant melanoma with ulceration and no clear surgical margins. She denied any additional surgical interventions and underwent to postoperative adjuvant radiotherapy. Follow up 5 months after initial diagnosis revealed no evidence of local recurrence or distant metastasis. The prognosis of vaginal primary malignant melanoma is very poor despite treatment modality, because most of the cases are diagnosed at advanced stage. Particularly patients with no clear surgical margins and tumor size >3 cm needed postoperative adjuvant radiotherapy.

## 1. Introduction

Vaginal primary malignant melanoma (VPMM) is a rare and very aggressive tumor [1, 2]. It accounts for 0.3–0.8% of all malignant melanomas, 2–5% of female genital tract melanomas, and less than 3% of all vaginal malignancies [1–3]. About 250 cases have been reported in the English literature [1, 2].

The estimated incidence of VPMM is 0.026/100,000 women per year [2, 3]. It most commonly occurs in postmenopausal women, with a mean age of 57 years [4–6]. There are no significant differences in VPMM incidence, between various racial or ethnic groups [3, 7].

The precise etiology of VPMM is relative unknown [8]. It is thought that VPMM arises from melanocytes present in the vaginal epithelium [8, 9]. However, it is obvious that ultraviolet radiation is not the causal factor in VPMM [7].

Our aim is to present a case of VPMM that underwent a wide local excision and postoperative adjuvant radiotherapy.

## 2. Case Presentation

The patient, an 80-year-old, gravida 3, para 2 postmenopausal Greek woman, presented to the Department of Obstetrics and Gynecology of the University of Patras Medical School with a complaint of abnormal vaginal bleeding. Her medical history included hypertension and diabetes mellitus. She had menopause at the age of 50. Her surgical history was unremarkable. Her family history revealed no evidence of cancer among the first-degree relatives.

On gynecologic examination there was a pigmented, raised, ulcerated, and irregular lesion 5 × 4.5 cm in the upper third of anterior vaginal wall. There were no palpable inguinal lymph nodes, and the rest of pelvic examination was normal. Preoperative computer tomography (CT) of the abdomen and pelvis, abdominal ultrasound (U/S), chest X-ray, intravenous pyelography (IVP), colonoscopy, and urethrocystoscopy was normal.

**Figure 1:**
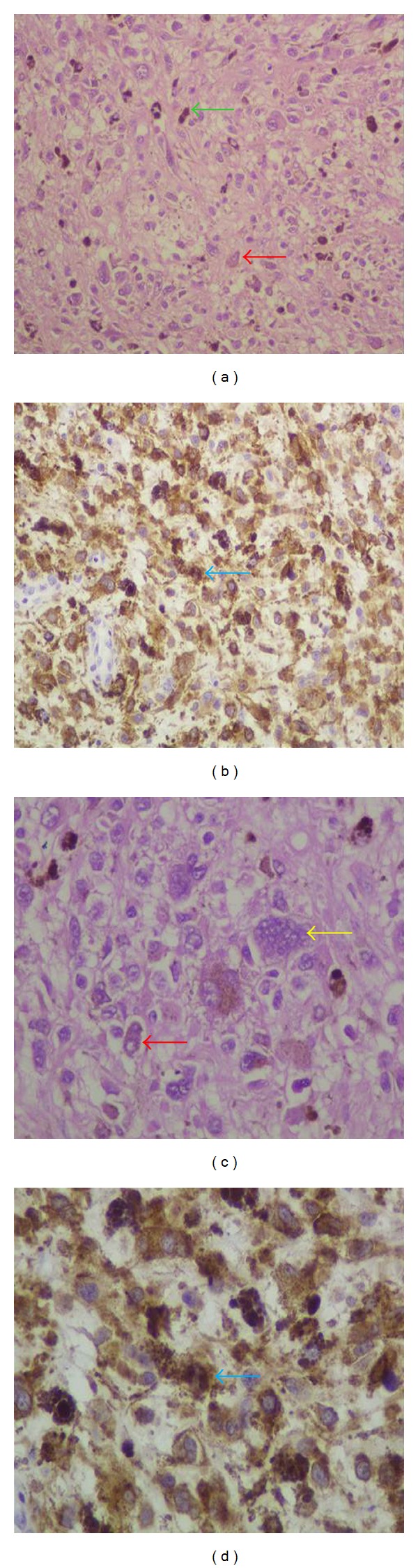
(a, b) Histologic details from tumor. The predominant cell type was epithelioid (red arrows) and not spindle cell. There were also multinuclear giant cells (yellow arrow) and abundant melanin deposition (green arrow) (H&E (a) ×200, (b) ×400). (c, d) Immunophenotype of tumor cells. The neoplastic cells expressed tyrosinase (blue arrows) (Streptavidin peroxidase (c) ×200, (d) ×400).

**Figure 2:**
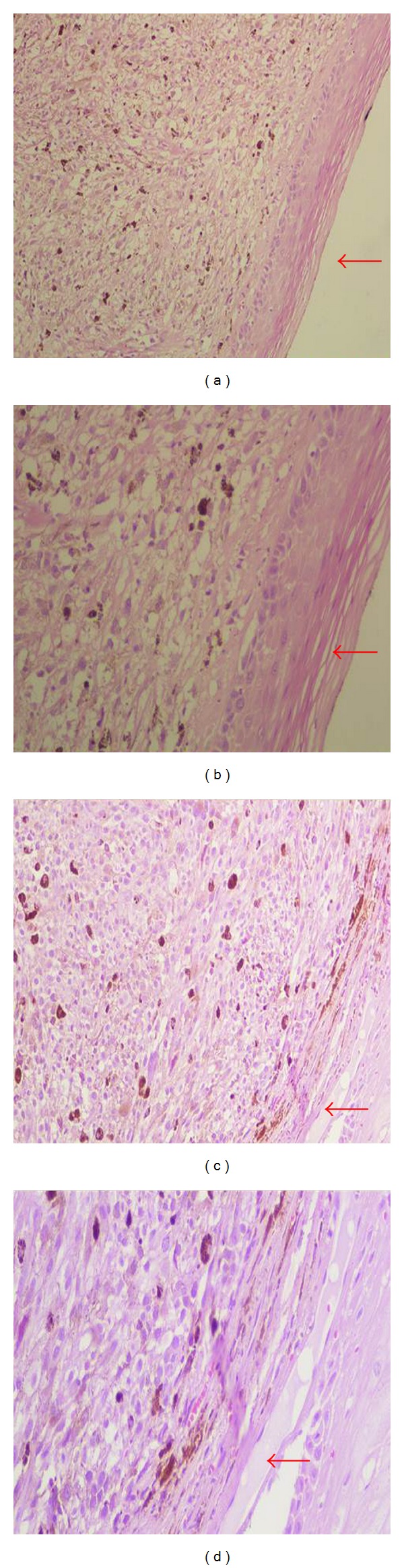
Tumor and vaginal epithelium. The arrows in (a, b) point the squamous cell epithelium of the vagina. The arrows in (c, d) point out the ulceration that tumor caused in the vaginal epithelium (H&E (a, c) ×200, (b, d) ×400).

**Figure 3:**
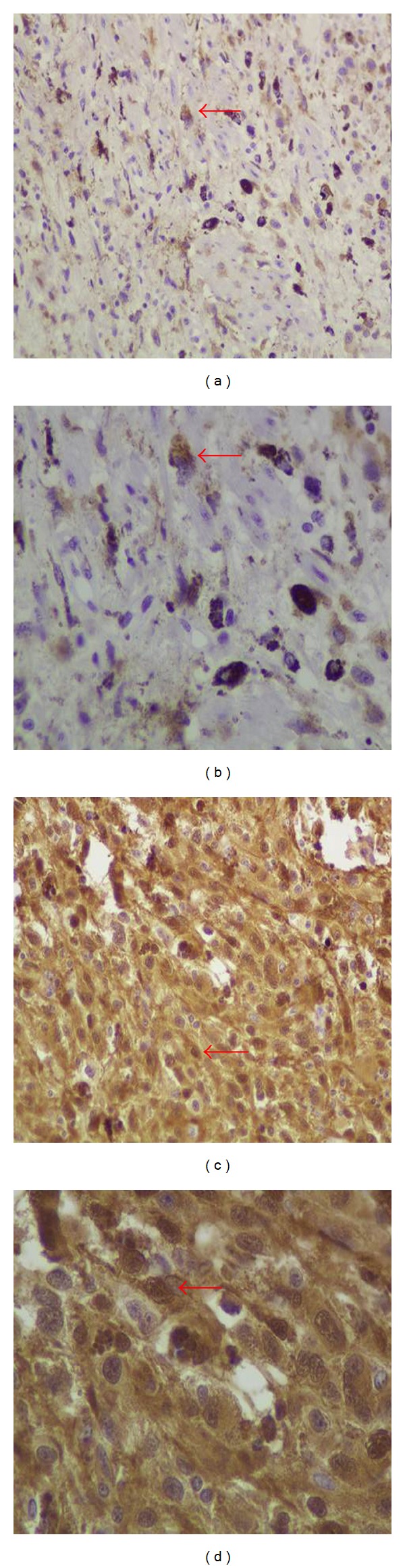
Immunophenotype of tumor cells. The neoplastic cells expressed melan A ((a, b) red arrows) and S-100 ((c, d) red arrows) (Streptavidin peroxidase (a, c) ×200, (b, d) ×400).

**Figure 4:**
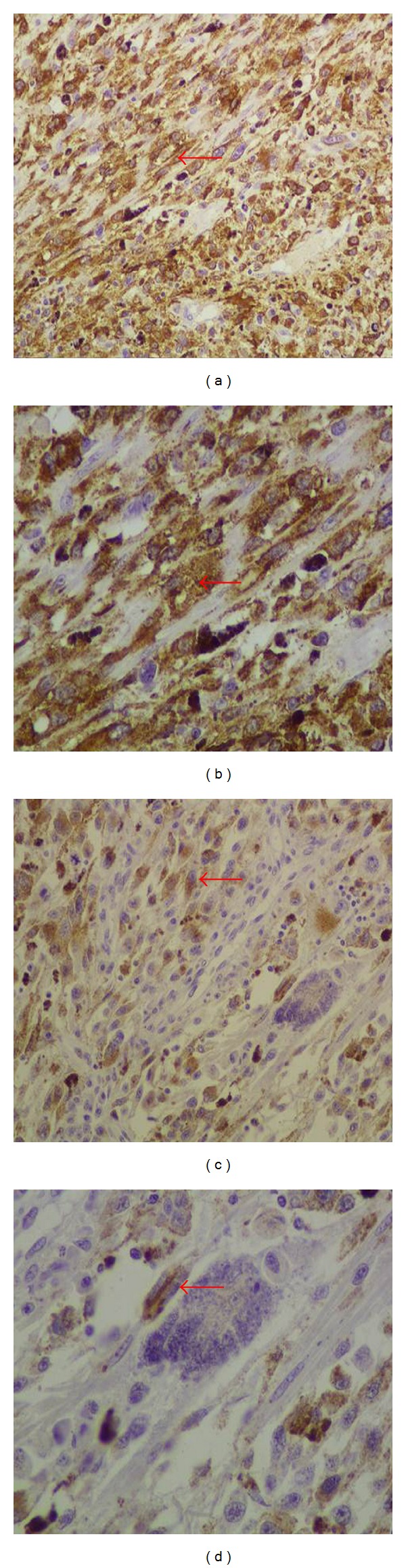
Immunophenotype of tumor cells The neoplastic cells expressed HMB45 ((a, b) red arrows) and microphthalmia transcription factor (MITF) ((c, d) red arrows) (Streptavidin peroxidase (a, c) ×200, (b, d) ×400).

She underwent a wide local excision of the lesion. The histopathology showed a malignant neoplasm consisting of tumor cells mainly with epithelioid and less with spindle cell morphology (Figure 1). There was abundant deposition of melanin and presence of multinuclear giant cells (Figure 1). In addition, the tumor caused ulceration of the squamous cell vaginal epithelium (Figure 2). The histologic diagnosis was confirmed by positive immunostaining. Tumor cells expressed tyrosinase, melan A, S-100 protein, HMB-45, and microphthalmia transcription factor (MITF) (Figures 1, 3, and 4). However, tumor cells did not express a-SMA, epithelial membrane antigen (EMA), and cytokeratin E3. The final diagnosis was vaginal primary malignant melanoma with ulceration and no clear surgical margins.

The patient denied any additional surgical interventions and underwent postoperative adjuvant radiotherapy. She received only high dose rate brachytherapy (HDRB) with ^192^Ir due to age, performance status, and comorbidities. The entire length of vagina was treated with HDRB in five weekly outpatient fractions using a vaginal cylinder 3 cm in diameter. Relying on the radiobiology of cutaneous malignant melanoma (CMM), a dose of 8 Gy given to a depth of 0.5 cm into the vaginal mucosa was administered with each treatment, for a total dose of 40 Gy [10].

Followup 5 months after initial diagnosis, with CT of the abdomen and pelvis, abdominal U/S, chest X-ray, IVP, colonoscopy, and urethrocystoscopy, revealed no evidence of local recurrence or distant metastasis.

## 3. Discussion

The precise histogenesis of VPMM is relatively unknown [8]. Probably VPMM arises from melanocytes located aberrantly in vaginal epithelium [8, 9]. Those melanocytes can be found in the basal layer of vaginal epithelium in 3% of healthy women [11]. It is thought that active junctional changes are the initial stage of development in malignant mucosal melanomas [12].

Although VPMM might arise anywhere, it is primarily found in the lower one third (34%) and mostly on the anterior wall (38%) of the vagina [2, 4]. VPMM may be single or multiple and pigmented or nonpigmented [13]. Also, most of VPMMs are polypoid and ulcerated [8, 13]. However, nonpigmented VPMMs may have similar appearance with vaginal epithelial tumors [8, 13]. In our patient, the ulcerated and pigmented tumor is located in the upper third of anterior vaginal wall.

The most common symptoms and signs in patients with VPMM are vaginal bleeding (80%), vaginal discharge (25%), palpable vaginal mass (15%), and pain (10%) [4, 8, 14, 15]. The main symptom of our patient was abnormal vaginal bleeding.

Epithelioid is the most common histologic cell type of VPMM (55%) [6, 8, 13]. Other less common histologic cell types of VPMM are spindled (17%) and mixed (28%) [6, 8, 13]. FIGO staging system for vaginal cancer is not suitable for VPMM, because it does not incorporate tumor size and regional lymph node status [2].

Although there are several treatment options for VPMM, an appropriate and effective treatment protocol has not been defined yet [6]. In those patients surgery remains the primary treatment of choice [2, 6]. The spectrum of surgery ranges from conservative (wide local excision) to radical (vaginectomy and pelvic exenteration) [2, 6]. If wide local excision with clear margins is possible, the role of radical surgery as primary treatment for VPMM remains unjustified [2, 6]. However, if local excision is not possible, pelvic exenteration may be reasonable [2]. Our patient underwent a wide local excision and denied any additional surgical interventions.

Lymph node dissection is not recommended in patients with VPMM, because the rate of lymph node metastasis is low [6]. Also the role of elective lymph node sampling in those patients remains controversial [2, 6]. Although it has no survival benefits, it leads to significant morbidity [6, 16]. Recently, the sentinel lymph node biopsy has gained popularity [6, 17].

Radiotherapy can be applied as primary treatment for patients who are unable or unwilling to have surgery [2, 6, 18, 19]. It can be applied preoperative as adjuvant treatment to reduce tumor size and enable a more conservative surgery [2, 6, 18, 19]. Also it can be applied postoperatively as adjuvant treatment for patients with incomplete tumor resection or with pelvic metastases [2, 6, 18, 19]. Our patient underwent a postoperative intravaginal HDRB, as she had no clear surgical margins, and denied any additional surgical intervention.

Dacarbazine (DTIC) has been the standard of care for many years in patients with advanced stage CMM, with response rates of 7.5% to 12.1% [20]. However, the role of chemotherapy in patients with advanced stage VPMM has not been established [21].

Immunotherapy with interferon (IFN) or interleukin-2 (IL-2) confers survival benefits in patients with VPMM at high risk for recurrence, but toxicity is important [15, 22–24]. IFN has been associated with the generation of autoantibodies and the induction of autoimmune disorders [25]. Immunotherapy has very low activity against metastatic or recurrent CMM [23, 24]. The combination of IFN and IL-2 is superior to IL-2 alone [26]. Our patient did not receive immunotherapy due to age, comorbidities, and risk of toxicity.

The combination of chemotherapy and immunotherapy (biochemotherapy) in patients with advanced stage CMM is associated with an increased response rate [24]. However, it has the disadvantage of increased toxicity [24]. Also it has no survival benefits, although it clearly improves response rates [24]. The role of biochemotherapy in patients with advanced stage VPMM has not been established [27].

VPMM is a very aggressive tumor, and most patients are diagnosed at advanced stage [1, 15, 22, 28]. This might be due to delayed diagnosis and the rich vascular and lymphatic network of the vaginal mucosa [4, 6, 14]. Those factors contribute to early tumor spread and development of metastases [6].

Despite treatment modality, 5-year survival in patients with VPMM ranges from 8.4% to 17.5% [1, 4, 6, 9]. Tumor size (<3 cm) is the most important prognostic factor, whereas tumor thickness is only a weak predictor of survival [4]. Many patients with VPMM have local recurrences in the pelvis and distant metastases in the lungs, liver, bones, and brain [4, 8]. Most of the patients with distant metastasis also have a concomitant local recurrence in the pelvis [4]. Our patient had tumor size >3 cm, and this is a dismal prognostic factor. However, 5 months after initial diagnosis, she has no evidence of local recurrence or distant metastasis.

It is obvious that the prognosis of VPMM is very poor despite treatment modality, because most cases were diagnosed at advanced stage [1, 15]. Also its prognosis is much more unfavourable, compared with other vaginal malignancies and CMM [8]. Particularly in patients with no clear surgical margins and tumor size >3 cm needed postoperative adjuvant radiotherapy.
